# Large-scale synthesis of functional single-atom catalysts

**DOI:** 10.1038/s42004-023-00834-4

**Published:** 2023-02-22

**Authors:** Yuhua Liu, Wei Zhang

**Affiliations:** Key Laboratory of Automobile Materials MOE, and School of Materials Science & Engineering, and Jilin Provincial International Cooperation Key Laboratory of High-Efficiency Clean Energy Materials, and Electron Microscopy Center, Jilin University, Changchun, 130012 China

## Abstract

Economical and high-efficiency synthesis of single-atom catalysts is a tremendous challenge hampering their large-scale industrialization, which is mainly attributed to the complex equipment and processes necessary for both top-down and bottom-up synthesis methods. Now, a facile three-dimensional printing approach tackles this dilemma. From a solution of printing ink and metal precursors, target materials with specific geometric shapes are prepared with high output, directly and automatically.

Single-atom catalysts (SACs) have become an emerging class of materials in the field of catalysis due to their 100% atomic utilization, controllable coordination environment, quantum size effects, unique electron-geometry structure and, ultimately, their excellent catalytic performance^1^. However, both the process of dimensionality reduction from bulk materials to single atoms, as well as the process of synthesis of host materials with subsequent high-temperature pyrolysis to prepare single atoms, involve complex wet-chemical steps. Dynamic assembly processes of SACs vary with experimental parameters; notorious sintering steps can lead to the formation of clusters and/or SACs. Similar synthetic challenges also exist for zero-dimensional quantum dot materials^2,3^. To increase production volume and effective loadings of SACs, efforts have been concentrated on preparing SACs precursors, such as by regulating ligand environment and construction of substrate defects^4,5^. Some improved mechanical methods have been applied to replace complicated wet-chemistry processes, such as mechanochemical abrasion, high-temperature-shockwave and laser-irradiation syntheses^6–9^. Various strategies to improve the loadings of SACs, such as defect engineering, coordinated design, and space restriction, have been reported^10^. While these methods have greatly improved the yield of SACs syntheses—marking a big step towards the goal of promoting SACs from laboratory to industrial production—cost-effectiveness, green chemistry, and convenience remain key factors of concern.

Now, Prof. Shi-Zhang Qiao from the University of Adelaide, Australia, together with an international team of researchers, reported a universal three-dimensional (3D) printing approach for preparing a library of SACs (10.1038/s44160-022-00193-3)^11^. “3D printing allows the customization of geometric designs from millimeters to meters, which is vital for industrial applications,” says Prof. Qiao. “The combination of 3D printing and single-atom catalysts provides a promising but simplified way to manufacture SACs at different scales.” The strategy is realized by mixing natural polymer printing inks and metal precursor solutions to produce centimeter-sized SACs precursor materials directly. The natural polymers are then carbonized, and SACs are simultaneously obtained via this heat treatment step (Fig. 1A). Thereby, the metal atoms are dissolved and undergo dynamic evolution processes^12^, including liquid metal agglomeration, dispersion, re-aggregation and sublimation, and then, captured by the carbonized carrier, a single-atom coordination structure is formed. The metallic atoms are confirmed as being atomically dispersed on the carbon substrate, as shown by the group through high-resolution high-angle annular dark-field scanning transmission electron microscopy (HAADF-STEM) and extended X-ray absorption fine structure (EXAFS) characterizations (Fig. 1B, C). Central atom type (M), coordination environment, as well as loading of the catalyst, can be varied. Qiao and team have selected different transition metal phthalocyanine and acetylacetone as precursors to prepare SACs with M–N–C and M–O–C coordinations, respectively. The M–O coordination can also be changed to a M–O–Cl coordination through subsequent hydrochloric acid treatment (Fig. 1D). By expanding or reducing the volume of the metal precursor, the loadings of single-atom sites can easily be regulated. This confirms the method’s versatility and offers an opportunity for SACs to meet the needs of a wide range of industrial applications. The synthesized catalysts are used for the reduction reaction of nitrate, attesting to the great potential of the manufacturing method in the field of electrocatalysis.

**Fig. 1:**
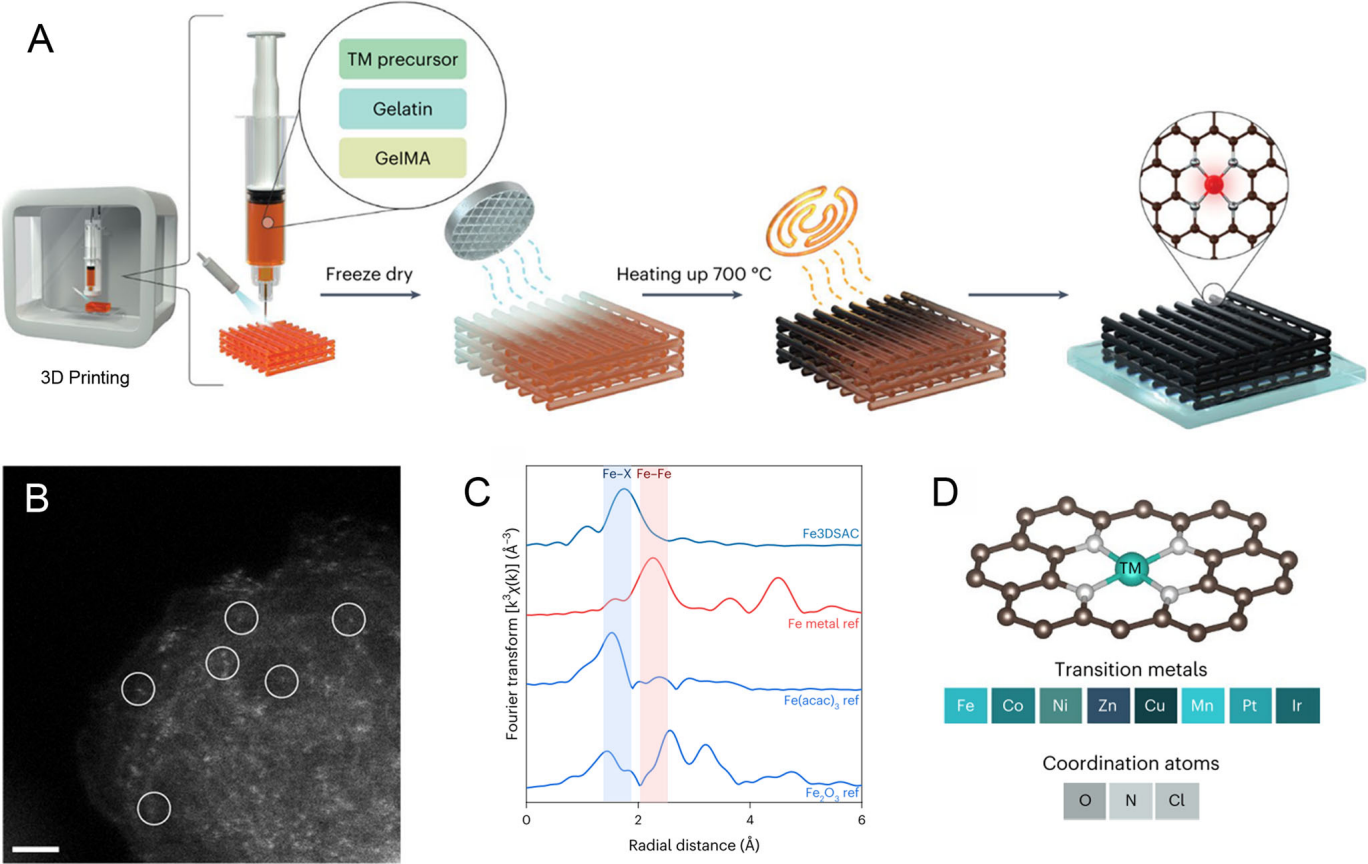
Preparation and characterization of single-atom catalysts. **A** Synthetic route of SACs by 3D printing strategy. **B** High-angle annular dark-field scanning transmission electron microscopy (HAADF-STEM) image of Fe SACs. Scale bar, 2 nm. **C** Extended X-ray absorption fine structure (EXAFS) characterization of Fe SACs. **D** Structure diagram of SACs with adjustable central atom and coordination environment. Reprinted with permission from Springer Nature: Nat. Synth. Copyright 2023.

This study illustrates the versatility and convenience of 3D printing and the technique’s suitability to reduce the cost of large-scale manufacturing of SACs. “3D-printed SACs pave the way to a scalable and sustainable production of valuable fuels and chemicals,” says Qiao. It is of great guiding significance to explore the structure–function relationship at the atomic level and gain mechanistic insight into functional materials. We hope the team’s efforts encourage the community to look closely at 3D-printed mono- and bimetallic SACs towards greater selectivity for catalytic reactions. One could envision this study as the starting point for a universal approach for a given material to be transformed into a pool of functionalized single atoms towards an even more comprehensive range of advanced functional materials.
